# Outbreak of Haff Disease along the Yangtze River, Anhui Province, China, 2016

**DOI:** 10.3201/eid2612.191186

**Published:** 2020-12

**Authors:** Huilai Ma, Jiabing Wu, Wei Qin, Chao Lin, Dan Li, Bing Zha, Qi Chen, Yan Ma, Tichao Zhou, Shicong Li, Lei Gong, Wanwan Ma, Dafang Ge, Zhouxiang Cheng, Jian Chen, Qun Li

**Affiliations:** Chinese Center for Disease Control and Prevention, Beijing, China (H. Ma, W. Qin, C. Lin, D. Li, Q. Chen, Y. Ma, T. Zhou, S. Li, Q. Li);; Anhui Center for Disease Control and Prevention, Hefei, China (J. Wu, L. Gong, W. Ma);; Lu’an Center for Disease Control and Prevention, Lu’an (W. Qin); Wuhu Center for Disease Control and Prevention, Wuhu, China (C. Lin, Z. Cheng);; Ma’anshan Center for Disease Control and Prevention, Ma’anshan (B. Zha, D. Ge, J. Chen)

**Keywords:** Haff disease, rhabdomyolysis, outbreak, crayfish, toxin, epidemiology, food safety, China

## Abstract

We investigated a large outbreak of Haff disease that occurred along the Yangtze River in Anhui Province, China, in 2016. Of the 672 cases identified during the outbreak, 83.3% (560/672) occurred in Wuhu and Ma’anshan. Patients experienced myalgia (100%) and muscle weakness (54.7%). The mean value of myoglobin was 330 + 121.2 ng/mL and of serum creatine kinase 5,439.2 + 4,765.1 U/L. Eating crayfish was the only common exposure among all cases; 96.8% (240/248) of implicated crayfish were caught on the shores of the Yangtze River or its connected ditches. Mean incubation period was 6.2 + 3.8 hours. This case–control study demonstrated that eating the liver of crayfish and eating a large quantity of crayfish were associated with an increased risk for Haff disease. The seasonal increases in crayfish population along the Yangtze River might explain the seasonal outbreaks of Haff disease.

Haff disease is an unexplained rhabdomyolysis that occurs within 24 hours after consumption of certain types of freshwater or saltwater fish (*1*,*2*). It was first reported in 1924 in the vicinity of Königsberg along the Baltic coast near Frisches Haff (*1*–*3*). Over the next 9 years, an estimated 1,000 persons were affected by similar outbreaks, occurring seasonally in the summer and autumn in this area (*3*). Although subsequent outbreaks were identified in several other countries, such as Sweden (*4*), the former Soviet Union (*5*), Brazil (*6*,*7*), Japan (*8*), and China (*9*,*10*), the etiology has not yet been determined. An unidentified heat-stable toxin similar to cyanotoxins or palytoxin, but primarily myotoxic and not neurotoxic, is thought to be the cause of Haff disease (*1*); however, evidence supporting this hypothesis has been scant.

In July 2016, the number of rhabdomyolysis cases reported to the National Foodborne Disease Surveillance System (NFDSS) in China dramatically increased in Anhui Province compared with previous years. Most of the cases were reported in Wuhu and Ma’anshan, cities in Anhui Province in eastern China. Epidemiologic features were compatible with Haff disease (*3*,*6*). Preliminary investigation implicated crayfish as the vector. On August 5, the number of cases surpassed 200, prompting an emergency investigation by the Chinese Field Epidemiology Training Program, together with the Anhui Province Center for Disease Control and Prevention (CDC). The objectives of the investigation were to describe the epidemiologic and clinical characteristics, trace back the implicated vectors, identify possible risk factors, and recommend control measures.

## Methods

### Case Definition and Finding

We defined a case of rhabdomyolysis as any person with elevation in creatine kinase (CK) value plus clinical manifestations of myalgia or limb weakness (*10*,*11*). We defined a Haff disease case as illness in any person with acute onset of rhabdomyolysis after ingestion of freshwater fish or seafood within 24 hours in Anhui Province during June–August 2016. We searched for physician-diagnosed rhabdomyolysis cases from the NFDSS, an internet-based, passive surveillance system for foodborne diseases searchable by food source in China. We also reviewed the outpatient and inpatient medical records in hospitals in Wuhu and Ma’anshan during the outbreak period to search for potential rhabdomyolysis cases.

Local Anhui Province CDC staff or Chinese Field Epidemiology Training Program trainees interviewed all rhabdomyolysis case-patients, either in-person or by telephone, using a structured questionnaire. Information collected included age, sex, date of onset, disease duration, clinical symptoms, potential risk factors (e.g., food, drugs, alcohol consumption, intense exercise, allergy history, underlying chronic illness), and the quantity of crayfish consumed. The researchers also obtained laboratory test findings from hospital medical records. They applied the Haff disease case definition to rhabdomyolysis cases to identify Haff disease cases and collected blood and urine specimens from Haff disease case-patients for further analysis.

In total, 673 rhabdomyolysis cases were identified in Anhui Province during June–August 2016. Of these, 99.9% (672/673) were compatible with the definition of Haff disease. All but 1 patient consumed cooked crayfish before symptom onset. The patient who did not eat cooked crayfish was a steelworker who had been working in the factory before onset, suggesting his illness might have been caused by heatstroke.

### Case–Control Study

Although nearly all Haff disease case-patients ate cooked crayfish, we did not know what percent of persons who did not become ill also ate meals containing crayfish, given that crayfish were widely available during June–August 2016. Therefore, we conducted a matched case–control study to assess the association between eating crayfish and Haff disease. Cases in this study were persons who met the definition for Haff disease, had shared a meal with someone else before symptom onset, and consented to participate in the study. We identified >1 control per case; controls were selected among persons who shared the suspected meal of exposure with the case-patient before symptom onset. Controls had no clinical symptoms compatible with rhabdomyolysis and consented to participate in the study. Persons with other illnesses (e.g., fever, cold, injury, etc.) were disqualified as controls. In total, 67 cases and 108 controls were enrolled in the case–control study. Trained investigators conducted telephone-based interviews August 7–15, 2016, using a standardized questionnaire.

### Traceback of Food and Environmental Investigation

For all Haff disease cases, we conducted a traceback investigation for the source of the implicated food by interviewing case-patients, restaurant owners, fishermen, and crayfish sellers. We conducted an environmental investigation of potential contamination along the distribution chain or unusual events during the outbreak period. We investigated the restaurants where case-patients had a meal before onset to find out where the crayfish came from and how they were cooked. We also visited crayfish farms, settings where crayfish were caught, and factories along the Yangtze River to identify whether the implicated crayfish or the environment in which the crayfish were raised had been contaminated.

### Data Analysis

We performed statistical analysis using SPSS Statistics 20 (IBM, https://www.ibm.com). We compared cases and controls by χ^2^ test. Significant risk factors (p<0.05) in the χ^2^ tests were included in a multivariate Cox proportional hazard model to determine the odds ratio (OR) and 95% CI for the potential risk factors associated with Haff disease. All of the p values were 2-sided, and p<0.05 was considered significant.

## Results

### Confirmation of the Outbreak

In total, we verified 672 Haff disease cases in Anhui Province during June–August 2016. All cases occurred in 7 cities along the Yangtze River in Anhui Province; 83.3% (560/672) of the cases occurred in Wuhu (334 cases) and Ma’anshan (226 cases). We focused our investigation on the cases that occurred in Wuhu and Ma’anshan. Of the 560 case-patients in Wuhu and Ma’anshan, 495 (88.4%) completed the questionnaires; all 495 had consumed crayfish within 24 hours before symptom onset. The epidemic curve suggested a continuing common-source outbreak (Figure).

**Figure:**
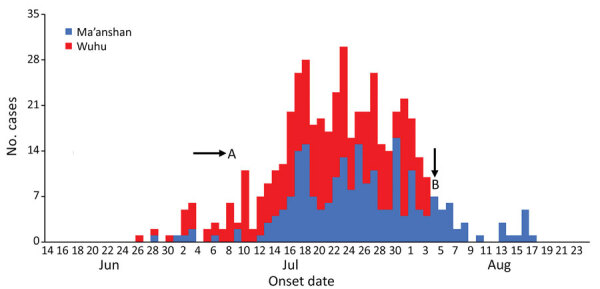
Outbreak of Haff disease in 2 cities along the Yangtze River, Anhui Province, China, 2016. A indicates period of heavy rainfall in Anhui Province; B indicates time at which local government warned residents not to eat crayfish.

### Descriptive Epidemiology

The outbreak started at the end of June, peaked in mid-July to early August, and lasted through August 17. Of the 495 case-patients, 197 (39.8%) were hospitalized; mean length of hospital stay was 7.3 ± 3.2 days. No deaths were reported. The mean age of the case-patients was 38.7 ± 13.5 years; 323/495 (65.3%) patients were female. Although cases were widely distributed in the 2 cities, 87.7% (434/495) were in residents from urban areas close to the Yangtze River. Each case-patient consumed a mean of 11.6 ± 6.1 crayfish pieces. The mean incubation period was 6.2 ± 3.8 hours.

### Clinical Characteristics

All 495 case-patients experienced myalgia that was local or diffuse, involving the back, waist, whole body, neck, limbs, and chest (Table 1). A total of 271/495 (54.7%) experienced muscle weakness. Additional symptoms included brown urine, dyspnea, vomiting, abdominal pain, dizziness, and headache. Symptoms of nerve paralysis and fever were rare. Acute renal failure was not observed.

**Table 1:** Clinical characteristics during Haff disease outbreak in Anhui Province, China, 2016

Symptoms	No. cases (%), N = 495
Myalgia	495 (100.0)
Back	241 (48.7)
Waist	194 (39.2)
Whole-body	186 (37.6)
Neck	186 (37.6)
Lower limbs	111 (22.4)
Upper limbs	89 (18.0)
Chest	59 (11.9)
Muscle weakness	271 (54.7)
Brown urine	99 (20.0)
Dyspnea	60 (12.1)
Headache	49 (9.9)
Abdominal pain	46 (9.3)
Diarrhea	32 (6.5)
Vomiting	29 (5.9)
Dizziness	25 (5.1)
Nausea	21 (4.2)
Nerve paralysis	11 (2.2)
Fever	1 (0.2)

### Laboratory Characteristics

We reviewed the laboratory test findings of blood and urine for some cases. The mean value of myoglobin was 330.0 ± 121.2 ng/mL, and mean CK level was 5,439.2 ± 4,765.1 U/L. In >80% of the cases, the levels of muscle-type CK and aspartate aminotransferase were abnormally elevated. In addition, 50.0% of case-patients were positive for urinary occult blood and proteinuria (Table 2).

**Table 2:** Laboratory test values from cases during Haff disease outbreak in Anhui, China, 2016*

Variables	Reference range	Median (range)	Mean + SD	No. cases	% Abnormal cases
Serologic test					
Myoglobin, ng/mL	<25	344.3 (25.0–500.0)	330.0 ± 121.2	97	100
CK, U/L	30–135	4,192.0 (165.0–17,470.0)	5,439.2 ± 4,765.1	191	100
CK-MM, U/L	0–25	79.0 (10.0–980.0)	161.2 ± 185.9	104	82.7
AST, U/L	14–36	73.0 (14.0–1,346.0)	164.6 ± 207.0	103	84.5
ALT, U/L	9–52	52.0 (20.0–515.0)	85.9 ± 88.2	101	49.5
LDH, U/L	313–618	684.0 (333.0–8,170.0)	1,161.0 ± 1,334.9	99	53.5
Cre, μmol/L	62–106	63.6 (34.6–108.3)	66.4 ± 14.5	102	44.1
Urea, mmol/L	2.5–6.1	5.7 (2.3–256.1)	8.1 ± 24.84	102	32.4
Cl^+^, mmol/L	98–107	104.70 (98.20–109.80)	104.46 ± 2.42	97	12.4
Ka^+^, mmol/L	3.6–5.0	3.95 (3.14–5.17)	3.97 ± 0.34	97	10.3
Ca^2+^, mmol/L	2.10–2.55	2.30 (2.07–2.65)	2.29 ± 0.13	47	8.5
Na^+^, mmol/L	137–145	139.20 (133.80–144.50)	139.25 ± 1.9	97	8.2
Urinalysis					
Proteinuria	−	No. positive results: 9		50.0	18
Urinary occult blood	−	No. positive results: 10		50.0	20

*ALT, alanine aminotransferase; AST, aspartate aminotransferase; CK, creatine kinase; CK-MM, muscle-type creatine kinase; LDH, lactate dehydrogenase; Cre, serum creatinine; Ka^+^, serum potassium, Na^+^, serum sodium; Cl^+^, Serum chloride; Ca^2+^, serum calcium.

### Case–Control Study

In the case–control study, 100% of the 67 cases and 93.3% (101/108) of controls ate crayfish during their shared meal (OR = ꝏ, 95% CI 0.92–ꝏ). We observed a significant dose-response relationship between the number of pieces of crayfish eaten and Haff disease (χ^2^ = 29.225; p<0.001) (Table 3). Further analysis showed that eating crayfish liver was associated with increased disease risk (OR = 4.0, 95% CI 1.2–12.7).

**Table 3:** Analysis of probable risk factors associated with Haff disease in case–control study, Anhui, China, 2016*

Variables	Cases, N = 67	Controls, N = 108	p value	Multivariable OR (95% CI)
Sex			0.033	
M	19 (28.4)	48 (44.4)		Referent
F	48 (71.6)	60 (55.6)		1.6 (0.9–2.7)
Mean age, y (SD)	37.3 (11.3)	39.4 (18.9)	0.227	NA
Consumption of crayfish (SD)			0.084	NA
No	0	7 (6.5)		
Yes	67 (100)	101 (93.5)		
No. crayfish consumed			<0.001	
1−9	27 (40.3)	84 (77.8)		Reference
10−19	26 (38.8)	12 (11.1)		2.4 (1.4–4.2)
>20	14 (20.9)	5 (4.6)		2.6 (1.3–5.1)
Ate liver of crayfish			<0.001	
No	3 (4.5)	32 (29.6)		Reference
Yes	64 (95.5)	76 (70.4)		4.0 (1.2–12.7)
Alcohol consumption			0.015	
No	51 (76.1)	97 (89.8)		Reference
Yes	16 (23.9)	11 (10.2)		1.6 (0.9–2.8)
Fish consumption†			0.648	—
No	64 (95.5)	100 (92.6)		
Yes	3 (4.5)	8 (7.4)		

*Values are no. (%) except as indicated. NA, not applicable; OR, odds ratio. SD, standard deviation. †Corrected χ^2^ value and p value for univariate analysis.

### Traceback and Environmental Investigation

Wuhu and Ma’anshan are located in the middle to lower reaches of the Yangtze River. Crayfish is a popular dish for residents of these 2 cities. Before the Haff disease outbreak, Anhui Province experienced heavy rainfall, which caused the largest flood disaster in decades. Consequently, rain or floodwater was retained in irrigation ditches and detention ponds for an extended time, and the amount of crayfish caught on the shores of the Yangtze River or its connected ditches was 5–10 times more during the outbreak period. However, no industrial or chemical contamination along the Yangtze River was reported.

The only common risk factor for all cases was eating crayfish, which were cooked thoroughly. We conducted a traceback investigation of the source for the implicated crayfish in Ma’anshan and Wuhu by interviewing persons in markets, restaurants, fisheries, and settings where crayfish were caught, as well as fishermen; we were able to trace 50.1% (248/495) of the implicated crayfish to their sources. Of these, 96.8% (240/248) were wild crayfish caught on the shores of the Yangtze River or its connected ditches. When we consulted with crayfish biologists, we found that the species of crayfish implicated during this outbreak was *Procambarus clarkii*.

### Public Health Measures 

Local governments issued a warning about the dangers of eating crayfish. In addition, public health departments instituted continuous surveillance and investigation of the outbreak.

## Discussion

The epidemiologic and traceback investigations of a large outbreak of Haff disease in Anhui Province, China, indicated that all case-patients consumed crayfish within 24 hours before symptom onset; the implicated crayfish were caught on the shores of the Yangtze River or its connected ditches. The case–control study revealed that eating the liver of crayfish was associated with an increased risk for disease; the risk increased as the quantity of crayfish eaten increased.

In China, the earliest reported outbreak of Haff disease was in Beijing in 2000 and involved 6 cases (*12*). An epidemiologic study revealed that all patients ate crayfish before onset, suggesting a link between crayfish and Haff disease (*12*). Although the literature shows that eating several species of fish, such as buffalo fish (*3*), salmon (*13*), freshwater pompano (*7*), marine boxfish (*8*), and pomfrets (*9*), could trigger Haff disease, almost all Haff disease cases in China were associated with eating crayfish (*2*). In recent years, Haff disease outbreaks have been reported in other cities in China (*14*–*19*). These outbreaks prompted the China CDC to conduct a thorough investigation of Haff disease. Crayfish have become a popular seafood for residents in central and eastern China, especially in June–September. Previous studies have reported that Haff disease shows a seasonal pattern, and outbreaks usually occur in the summer and fall months (*1*,*3*,*10*). Although a large Haff disease outbreak caused by eating freshwater pomfret occurred in October 2009 in southern China (*9*), most crayfish-related outbreaks (*10*,*20*,*21*), clusters (*18*,*22*), and sporadic cases (*16*) occurred predominantly in the summer. Seasonal crayfish harvest and consumption in June–September likely increases the opportunities for exposure, which may partially explain the seasonal pattern of Haff disease in China (*10*).

The most commonly reported clinical features in this outbreak were myalgia and muscle weakness, as well as abnormal levels of myoglobin and CK. Increased serum myoglobin concentration is the basis for early diagnosis of rhabdomyolysis (*23*); however, myoglobin concentrations tend to normalize within 6–8 hours following exposure. Thus, the window of opportunity for diagnosis is short (*24*). Of note, elevated myoglobin concentrations were observed in all case-patients who were tested in this study; this may be due to prompt medical care and timely laboratory testing in the hospital.

Rhabdomyolysis is a common life-threatening syndrome characterized by the injury of skeletal muscle resulting in the leakage of intracellular contents into the circulatory system (*25*). Patients with rhabdomyolysis usually experience myalgia, muscle weakness, raised serum CK, and brown urine (*24*). The etiologic spectrum of rhabdomyolysis is extensive, including crush injuries, ischemia, strenuous exercise, extreme body temperatures, drugs, toxins, infections, hereditary causes, and inflammatory or autoimmune muscle disease (*23*,*25*). A substantial number of patients may have no cause identified. We found that nerve paralysis and fever were rare symptoms, all crayfish were cooked thoroughly, and no industrial or chemical contamination was identified; therefore, this outbreak was unlikely to have been caused by infectious or chemical etiologies. Diaz et al. reported that an unidentified, heat-stable, algal toxin with primarily myotoxic rather than neurotoxic properties in seafood has been proposed as a cause of Haff disease (*1*); whether this toxin also exists in crayfish remains unknown.

Although many Haff disease cases have occurred in cities located in the middle to lower reaches of the Yangtze River, the association between Haff disease and crayfish caught from Yangtze River has not been elucidated in the published literature (*10*,*18*,*20*,*26*). In recent years, 3 other large Haff disease outbreaks have been reported in Nanjing and Tongling, 2 other cities located in the middle to lower reaches of the Yangtze River (*10*,*19*–*21*). The fact that these outbreaks all occurred in the middle to lower reaches of the Yangtze River suggests that crayfish could be their common etiology.

Studies using a mouse model have found that the hazardous substance from crayfish could cause rhabdomyolysis (*27*,*28*). This hazardous substance is specific to certain batches of crayfish. A dose-response relationship has also been observed. These findings in laboratory animals were consistent with the results of human epidemiologic investigation (*21*,*28*) and with our case–control study findings.

Our study had several limitations. First, because we lacked data on how many persons ate crayfish in the 2 study cities, we could not calculate the attack rates. Second, not all crayfish were traced back to their sources. Third, we were unable to conduct animal experiments to prove causation.

In conclusion, during this outbreak, the risk for Haff disease was associated with eating crayfish along the Yangtze River. The etiology of Haff disease remains elusive due to lack of knowledge of the underlying disease mechanism of rhabdomyolysis. Our findings might help researchers isolate the toxin that causes this disease.

## References

[1] Diaz JH. Global incidence of rhabdomyolysis after cooked seafood consumption (Haff disease). Clin Toxicol (Phila). 2015;53:421–6. 10.3109/15563650.2015.1016165 25789572

[2] Chan TY. The emergence and epidemiology of Haff disease in China. Toxins (Basel). 2016;8:359. 10.3390/toxins8120359 27916937 PMC5198553

[3] Buchholz U, Mouzin E, Dickey R, Moolenaar R, Sass N, Mascola L. Haff disease: from the Baltic Sea to the U.S. shore. Emerg Infect Dis. 2000;6:192–5. 10.3201/eid0602.000215 10756156 PMC2640861

[4] Berlin R. Haff disease in Sweden. Acta Med Scand. 1948;129:560–72. 10.1111/j.0954-6820.1948.tb09326.x 18914341

[5] Sidorova LD, Ierusalimskaia LA, Valentik MF, Razenko TN, Bredikhin AV. [Kidney lesions in dietary and toxic paroxysmal myoglobinuria (Iuksovsk-Sartlansk disease)] [in Russian]. Ter Arkh. 1985;57:120–3. 4081987

[6] Bandeira AC, Campos GS, Ribeiro GS, Cardoso CW, Bastos CJ, Pessoa TL, et al. Clinical and laboratory evidence of Haff disease - case series from an outbreak in Salvador, Brazil, December 2016 to April 2017. Euro Surveill. 2017;22:30552. 10.2807/1560-7917.ES.2017.22.24.30552 28661391 PMC5479974

[7] dos Santos MC, de Albuquerque BC, Pinto RC, Aguiar GP, Lescano AG, Santos JH, et al. Outbreak of Haff disease in the Brazilian Amazon. Rev Panam Salud Publica. 2009;26:469–70. 10.1590/S1020-49892009001100012 20107699 PMC4066848

[8] Taniyama S, Sagara T, Nishio S, Kuroki R, Asakawa M, Noguchi T, et al. [Survey of food poisoning incidents in Japan due to ingestion of marine boxfish and their toxicity]. Shokuhin Eiseigaku Zasshi. 2009;50:270–7. 10.3358/shokueishi.50.270 19897955

[9] Huang X, Li Y, Huang Q, Liang J, Liang C, Chen B, et al. A past Haff disease outbreak associated with eating freshwater pomfret in South China. BMC Public Health. 2013;13:447. 10.1186/1471-2458-13-447 23642345 PMC3651293

[10] Chen Y, Yuan B, Xie G, Zhen S, Zhou Y, Shao B, et al. Outbreak of Haff disease caused by consumption of crayfish (*Procambarus clarkii*), Nanjing, Jiangsu Province, China. Food Control. 2016;59:690–4. 10.1016/j.foodcont.2015.06.031

[11] Bagley WH, Yang H, Shah KH. Rhabdomyolysis. Intern Emerg Med. 2007;2:210–8. 10.1007/s11739-007-0060-8 17909702

[12] Yuan Y, Chen QT. Clinical analysis of six cases with Haff disease after eating crayfish [in Chinese]. Zhonghua Yi Xue Za Zhi. 2001;81:1530–1.

[13] Langley RL, Bobbitt WH III. Haff disease after eating salmon. South Med J. 2007;100:1147–50. 10.1097/SMJ.0b013e3181583673 17984750

[14] Sun S. Investigation on a case of rhabdomyolysis caused by crayfish [in Chinese]. Zhi Ye Yu Jian Kang. 2011;27:788.

[15] Zhu L. Investigation of two crayfish cases related to rhabdomyolysis syndromes [in Chinese]. Yu Fang Yi Xue Lun Tan. 2015;21:700–3.

[16] Feng G, Luo Q, Zhuang P, Guo E, Yao Y, Gao Z. Haff disease complicated by multiple organ failure after crayfish consumption: a case study. Rev Bras Ter Intensiva. 2014;26:407–9. 25607271 10.5935/0103-507X.20140062 PMC4304470

[17] Gan L, Li Q, Gong NK. Two cases of rhabdomyolysis diagnosis caused by eating crayfish [in Chinese]. Journal of Jinzhou Medical University. 2015;36:111–2.

[18] Zhang B, Yang G, Yu X, Mao H, Xing C, Liu J. Haff disease after eating crayfish in east China. Intern Med. 2012;51:487–9. 10.2169/internalmedicine.51.6786 22382564

[19] Liu JJ, Yong H, Shenwei Q, Yixin H. Epidemiological investigation of crayfish related rhabdomyolysis in Tongling, 2016–2017 [in Chinese]. China Trop Med. 2018;18:899–903.

[20] Ma S, Xu C, Liu S, Hu Z, Liu W, Zhang J, et al. [Epidemiology characteristics of crawfish related rhabdomyolysis in Nanjing, 2016: a multicenter retrospective investigation] [in Chinese]. Zhonghua Wei Zhong Bing Ji Jiu Yi Xue. 2017;29:805–9. 28936956 10.3760/cma.j.issn.2095-4352.2017.09.008

[21] Guo B, Xie G, Li X, Jiang Y, Jin D, Zhou Y, et al. Outbreak of Haff disease caused by consumption of crayfish (*Procambarus clarkii*) in nanjing, China. Clin Toxicol (Phila). 2019;57:331–7. 10.1080/15563650.2018.1529318 30451016

[22] Yang WX, Fan KL, Leung LP. A cluster of patients with rhabdomyolysis after eating crayfish. CJEM. 2018;20(S2):S48–S50. 10.1017/cem.2017.391 28893338

[23] Warren JD, Blumbergs PC, Thompson PD. Rhabdomyolysis: a review. Muscle Nerve. 2002;25:332–47. 10.1002/mus.10053 11870710

[24] Cervellin G, Comelli I, Lippi G. Rhabdomyolysis: historical background, clinical, diagnostic and therapeutic features. Clin Chem Lab Med. 2010;48:749–56. 10.1515/CCLM.2010.151 20298139

[25] Khan FY. Rhabdomyolysis: a review of the literature. Neth J Med. 2009;67:272–83. 19841484

[26] He F, Ni J, Huang JA, Liu Y, Wu C, Wang J. Clinical features of Haff disease and myositis after the consumption of boiled brackish water crayfish: a retrospective study of 96 cases at a single centre. Intern Emerg Med. 2018;13:1265–71. 10.1007/s11739-018-1870-6 29737466

[27] Chen XF, Lin JW, Pan TM, Cao MJ, Shi CL, Cai QF, et al. Investigation of the hazardous substance causing crayfish-induced rhabdomyolysis via a mouse model, a hemolysis assay, and a cytotoxicity assay. Fish Sci. 2015;81:551–8. 10.1007/s12562-015-0856-9

[28] Huang Q, Zhao M, Wang FY, Tan JB, Chen BF, Li XQ, et al. Population epidemiological investigation of crayfish-related rhabdomyolysis syndrome and triggering experiments in mice [in Chinese]. Zhongguo Shipin Weisheng Zazhi. 2017;29:269–76.

